# Shear modulus reduction and damping ratios curves joined with engineering geological units in Italy

**DOI:** 10.1038/s41597-023-02412-8

**Published:** 2023-09-14

**Authors:** Iolanda Gaudiosi, Gino Romagnoli, Dario Albarello, Carolina Fortunato, Paola Imprescia, Francesco Stigliano, Massimiliano Moscatelli

**Affiliations:** 1 Istituto di Geologia Ambientale e Geoingegneria, Consiglio Nazionale delle Ricerche, Montelibretti, RM 00015 Italy; 2 Dipartimento per il Servizio Geologico d’Italia, Istituto Superiore per la Protezione e la Ricerca Ambientale, Rome, 00144 Italy; 3 Dipartimento di Scienze Fisiche, della Terra e dell’Ambiente, Università degli Studi di Siena, Siena, 53100 Italy

**Keywords:** Natural hazards, Civil engineering

## Abstract

Numerical simulations of seismic site response require the characterization of the nonlinear behaviour of shallow subsoil. When extensive evaluations are of concern, as in the case of seismic microzonation studies, funding problems prevent from the systematic use of laboratory tests to provide detailed evaluations. For this purpose, 485 shear modulus reduction, G\G_0_(*γ*) and damping ratio, D(*γ*) curves were collected from multiple literature sources available in Italy. Each curve was associated with the related engineering geological units considered in seismic microzonation studies. A statistical analysis of the data was carried out with the aim of shedding light on the significant difference between the laboratory classification of samples and the macroscopic/engineering geological one, provided during seismic microzonation studies. Since the engineering geological classification plays a prominent role in extensive site response evaluations, the outcomes of the present work may be of help at least when preliminary seismic response estimates are of concern. The dataset provides reference information that can serve as key data for large-scale hazard assessments worldwide.

## Background & Summary

Simulations of waves propagation are a recurrent practice for the quantification of the ground motion expected at a site. Since they are performed considering the uncertainties of one or more parameters that play a role in seismic hazard, they may be included among the probabilistic seismic hazard applications (PSHA). As reported in the existing literature, PSHA contemplate different levels of increasing complexity^1^. In level 0, a fully probabilistic seismic hazard estimate requires the convolution of the hazard defined on bedrock virtually ignoring site-specific information^2–4^, and of amplification factors derived from a Ground Motion Prediction Equations logic tree^5^. In the subsequent levels, fully probabilistic hazard estimates are retrieved adopting a site-specific approach, introducing single-station standard deviation values and validations at seismological networks^6^, or the convolution of the hazard curve on rock with the probability distribution of the amplification functions obtained from analytical soil response analyses. In this latter case, estimates on the variability of ground motion, S-waves velocities Vs and nonlinear properties associated with the local seismo-stratigraphical configuration are necessary^7^.

It is now worth noting that the geographical scale of the PSHA fits well with the scale of the seismic microzonation (SM) studies. Therefore, information obtained at large (local) or even regional scale (<1:25,000) through SM studies may be especially useful for PSHA. SM is a practice that is able to account for site effects at different levels of detail (known as 1st, 2nd or 3rd level), highlighting on 1:10,000 scale or more detailed specific maps the areas most prone to seismic hazard^8^. It is commonly accepted that SM is a tool of fundamental importance for land use, planning and to maintain engineering infrastructures^9,10^, although SM should be considered only the first step towards a comprehensive seismic hazard assessment of the total site-specific hazard. Starting from the results of extensive numerical modelling performed over 138 municipalities in central Italy^11^, the attempt to represent absolute estimates of the seismic hazard has already been followed by Mori *et al*.^12^. Also, Barani *et al*.^13^ provided another example in Italy of incorporating the results of 2nd level SM studies into probabilistic seismic hazard analysis.

Still further analyses should be performed, however, and advanced methods developed to extend the results over wider areas. This can be achieved using already available data such as the significant amount of morphological and/or geological-geotechnical data, which together may provide the possibility of defining appropriate proxies (Vs30) that are suitable to catch the site amplification, with a partially probabilistic/hybrid approach^14^. It should be noted that, in common practice, due to the fact that increasing the SM level is a function of the available economic resources, SM studies are often only qualitative (i.e. if the SM is conducted at a 1st level) or, if they contain quantitative estimates (i.e. in terms of amplification factors, if the SM is conducted at a 2nd or 3rd level), they do not provide synthetic parameters that express the local and reference hazard together computed probabilistically. Only at the building/structure scale, several analyses have been carried out by one-dimensional Monte Carlo ground response analyses using a fully probabilistic approach^15–17^.

Thus, the rationale behind this study rests on the proposition of a robust harmonization of the data available from SM studies to support the need for fully probabilistic seismic site-specific hazard studies, of which still today no example exists. Otherwise, it should be considered that in Italy up to 2,000 1st level SM studies are available (https://www.webms.it/servizi/stats.php) and the integration of the subsoil engineering geological model^18^ with with the association with Vs has already been attempted by Romagnoli *et al*.^19^.

One of the basic products of a SM study is an engineering geological map and the conceptual interpretation of the subsoil under investigation in terms of engineering geological units. This kind of information differs from that contained in a basic geological map because the latter does not represent the dynamic nature of the subsoil^20^.

Starting from the engineering geological setting, it is possible to aggregate samples used for laboratory tests to the same engineering geological unit: a dataset associated with this kind of engineering geological unit classification constitutes an untried method in existing research.

The paper is organized in three main parts. After a general description of the data and methods, statistics are shown and, finally, comparisons with literature data are discussed in detail.

## Methods

As the first step, shear modulus reduction, G\*G*_0_(*γ*) and damping ratio, D(*γ*) curves were collected and associated with the related engineering geological units considered in SM studies (eg-units hereafter). The goal of the engineering-geological classification proposed for Italian SM studies is to group together soils and rocks in two main categories, the “Cover terrains” and “Geological bedrock” units respectively, considering their geological and geotechnical properties or attributes in order to analyse the seismic local effects at urban municipality scale^21^. The Cover terrains units collectively represent all kinds of loose, incoherent and unconsolidated superficial deposits such as, gravel, sand, clay, and organic material that originated generally in the Quaternary era. These include slope deposits, terraced and recent alluvial deposits, terraced marine deposits, polygenic detritic coverings, ancient glacial deposits, lacustrine sediments, eluvial-colluvial and landslide deposits. The cover units are classified, according to the Unified Soil Classification System^22,23^ (Table 1), into coarse grain and fine-grained soils. The coarse grain soils consist of gravels (G) and sands (S). Each class is further subdivided into four units depending upon the grading and inclusion of other grain sized materials, combining the “G” or “S” acronyms with “W” for well graded, “P” for poorly graded, “M” for containing fine materials and “C” for clay binder. The coarse grain soils also contain anthropic deposits (RI). The fine-grained soils include silts and clays and are divided into three classes named with the acronyms “M” for inorganic silts and very fine sands, “C” for inorganic clays and “O” for organic silts and clays. These are combined, on the basis of their liquid limit and plasticity index, with the acronyms “L” for low plasticity and “H” for middle and high plasticity. The fine-grained soils also contain peat and other highly organic soils (PT). The designation of unconsolidated units refers to the dominant grain size of clastic material mixtures of different sizes. The Cover terrains are thus classified in 16 eg-units (Table 1). The Geological bedrock units consist of lithoid and consolidated deposits of geological formations, comprising weathered and fractured portions, classified following lithostratigraphic criteria, structural features and facies^19,24,25^. Examples of Geological bedrock units are limestones, sandstones, siltstone, dolomites, chert, marly calcareous and marly bedrock, pelitic and arenaceous bedrock, brecciated and conglomeratic bedrock. This category also includes 16 e-g units starting from 4 main types of rocks: lapideous rocks “LP” (e.g. limestone, dolomites), grainy cemented rocks “GR” (e.g. sandstones, conglomerates), cohesive over-consolidated rocks “CO” (e.g. over-consolidated clays) and deposits characterized by alternations of the contrasting lithotypes “AL” (e.g. flysch deposits). All the other units derive from these four main units. If they are stratified, the acronym “S” is added to form another 4 units (“LPS”, “GRS”, “COS” and “ALS”). If the previous 8 eg-units are fractured and/or weathered, the prefix “SF” is added to the beginning of the acronym (e.g. “SFLP”, “SFGRS”, “SFALS”, “SFCO”; Table 1). The Italian SM classification of the geological bedrock units considers the intact, stratified and weathered and/or fractured rock properties^21^.

**Table 1:** Engineering–geological classification adopted in SM studies by following Italian standards^21^.

eg-units	Cover terrain group	eg-units	Cover terrain group
**RI**	Terrains containing remains of human activity, anthropic deposits	**LP**	Lapideous rock
**GW**	Well sorted gravels, mixed gravels and sands	**GR**	Grainy cemented rock
**GP**	Non sorted gravels, mixed gravels and sands	**CO**	Cohesive over-consolidated rock
**GM**	Silty gravels, mixed gravels, sands and silts	**AL**	Alternations of lithotypes
**GC**	Clayey gravels, mixed gravels, sands and clays	**LPS**	Stratified LP
**SW**	Well sorted sands, mixed sands and gravels	**GRS**	Stratified GR
**SP**	Poorly sorted sands	**COS**	Stratified CO
**SM**	Silty sands, mixed sands and silts	**ALS**	Stratified AL
**SC**	Clayey sands, mixed sands and clays	**SFLP**	Fractured/weathered LP
**OL**	Organic silts, low plasticity organic silty-clays	**SFGR**	Fractured/weathered GR
**OH**	Middle plasticity organic clays, organic silts	**SFCO**	Fractured/weathered CO
**MH**	Inorganic silts, fine sands, diatomic silts	**SFAL**	Fractured/weathered AL
**ML**	Inorganic silts, fine silty-clayey sands, low plasticity clayey, silts	**SFLPS**	Fractured/weathered LPS
**CL**	Middle-low plasticity inorganic clays, gravel-sandy clays, silty clays	**SFGRS**	Fractured/weathered GRS
**CH**	High plasticity inorganic clays	**SFCOS**	Fractured/weathered COS
**PT**	Peat and organic soils	**SFALS**	Fractured/weathered ALS

All the G\*G*_0_(*γ*) and D(*γ*) curves were singularly regularized according to the procedure proposed by Yokota *et al*.^26^.

This latter allows the relationships between G\*G*_0_ and the strain *γ* to be found as the simplified formula in Eq. (1), and D as the simplified formula in Eq. (2). Substantially, G\*G*_0_ and D values are defined by means of the three constants: namely, *α* and *β* for G\*G*_0_ and *λ* for D:

1 $$\frac{G}{{G}_{0}}=\frac{1}{1+\alpha \cdot {\gamma }^{\beta }}$$

2 $$D={D}_{max}\cdot {e}^{\lambda \frac{G}{{G}_{0}}}$$

The constants *α*, *β* and *λ* are obtained through a double-step procedure of adaptation of the experimental data to the analytical linearized expressions of Eqs. (1, 2): firstly the parameters of Eq. (1) are obtained and then they are used to calibrate *λ*.

Curves were regularized up to a value of the *γ* level of 0.0001%. The raw data was archived As-Is in the original pdf file, while curves were each individually regularized using a codified procedure. Before defining parameters of the Yokota modeling adaptation, points recognized outliers were manually deleted. A further and parallel regularization was also performed considering all the curves for each eg-unit with the aim of representing the behaviour of soils in macroscopic terms.

## Data Records

The collection, available at the link: 10.5281/zenodo.8134979^27^, has been carried out nationwide considering the available data from SM studies, public databases and published works, according to European Commission principles^28^.

Primarily, in order to have a Findable, Accessible, Interoperable, Re-usable (so called FAIR) dataset (developed according to the European Open Science Cloud - EOSC policies, https://ec.europa.eu/research/openscience/index.cfm?pg=open-science-cloud), each set of laboratory test results has been saved in a standard file. The dataset will be useful for accomplishing the following purposes: to access and interoperate research data throughout web-accessible services (for instance, by means of the webpage: https://www.webms.it/servizi/catalog.php) and guarantee public access to subsoil information in the perspective of data integration with already existing web-based databases (i.e., European Geotechnical Database service – EGD, http://egd-epos.civil.auth.gr/; the New Zealand Geotechnical Database – NZGD, https://www.nzgd.org.nz/). This approach will ensure the continuous maintenance of the dataset, which can be updated every time new information is available.

Each file in the dataset is described in its accompanying metadata file, which can be seen as a complementary footnotes sheet. The metadata file contains the following information:

- Rootfilename: basename of the raw archived file;
- Macroarea: name of the macroarea (region or area) of the SM study where the sample was collected;
- Municipality: the municipality where the sample was taken;
- Type of laboratory test;
- Depth *top* and bot (m): depth of the top and bottom sampling computed from the surface level;
- *γ* (kN\*m*^3^): unit weight;
- WL (%): water content at the liquid limit;
- PI (%): Plasticity Index;
- USCS code: code according to the USCS classification;
- eg-unit SM: code of the eg-unit retrieved from SM study;
- X and Y coordinates from a WGS84/UTM-33N datum;
- Ref: link or references to the data source.
- Namely, the dataset consists of 485 G\*G*_0_(*γ*) and D(*γ*) curves obtained from:
- the third level of the SM studies carried out following the 2016–2017 Central Italy seismic sequence (https://sisma2016data.it/), in which the dynamic behaviour of silty and clayey soils was first studied by Ciancimino *et al*.^29^;
- SM studies carried out in the Emilia Romagna region^30^;
- SM studies of Roma Palatino^16,31,32^;
- SM studies of Nocera Umbra^33^;
- SM studies carried out following the 2009 L’Aquila earthquake (MS AQ Working Group^34^);
- SM studies carried out on the eastern flank of the Mount Etna volcano following the 2002 Santa Venerina earthquake (Protezione Civile Catania Working Group^35^ and Cavallaro *et al*.^36^);
- the database VEL (Valutazione degli Effetti Locali) project, devoted to seismic risk mitigation of the Toscana region (http://150.217.73.23/BancaDatiVEL/project).

**Fig. 1:**
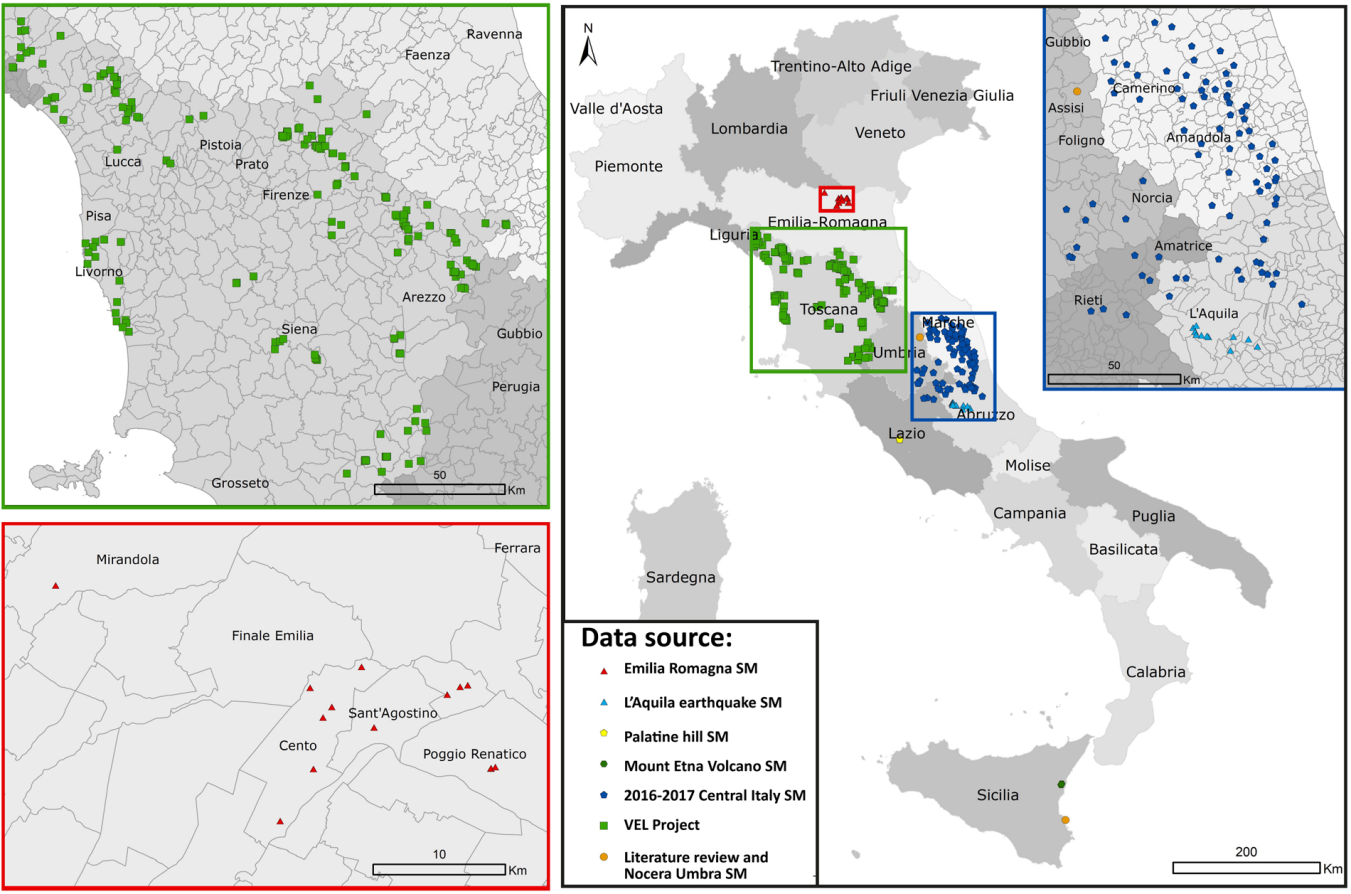
Location of the investigated sites.

The geographic location of the sites where laboratory tests were collected is shown in Fig. 1.

**Fig. 2:**
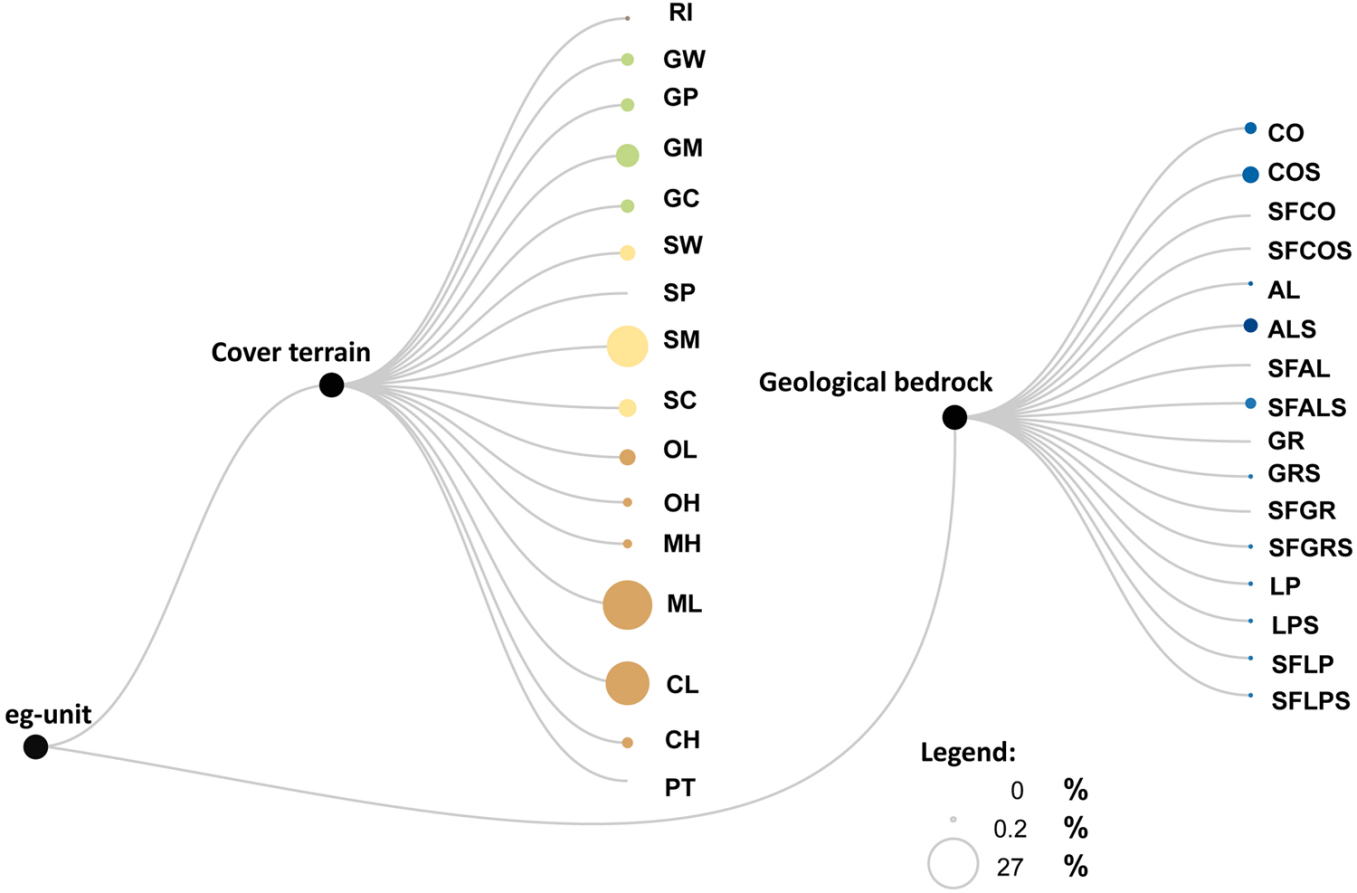
Dendrogram of the number of available samples. Each node represents a single eg-unit. The size of the nodes is proportional to the number of available samples.

We analysed the experimental G\*G*_0_(*γ*) and D(*γ*) curves obtained from different types of geotechnical laboratory tests: Double Specimen Direct Simple Shear, DSDSS; Resonant Column, RC; Cyclic Triaxial, TXC; Cyclic Torsional Test, CT; Cyclic Torsional Shearing, CTS; Resonant Column and Cyclic Torsional Test, RCT. In several sites, for each sampled layer, different laboratory tests were performed to enlarge the range of deformations analysed. In these cases, the results are reported in the dataset in separate rows. Figure 2 graphically visualises the similarities among available samples: samples were taken mostly in unconsolidated clastic deposits of cover terrain units, although about 10% of the tests were carried out for geological bedrock units.

**Fig. 3:**
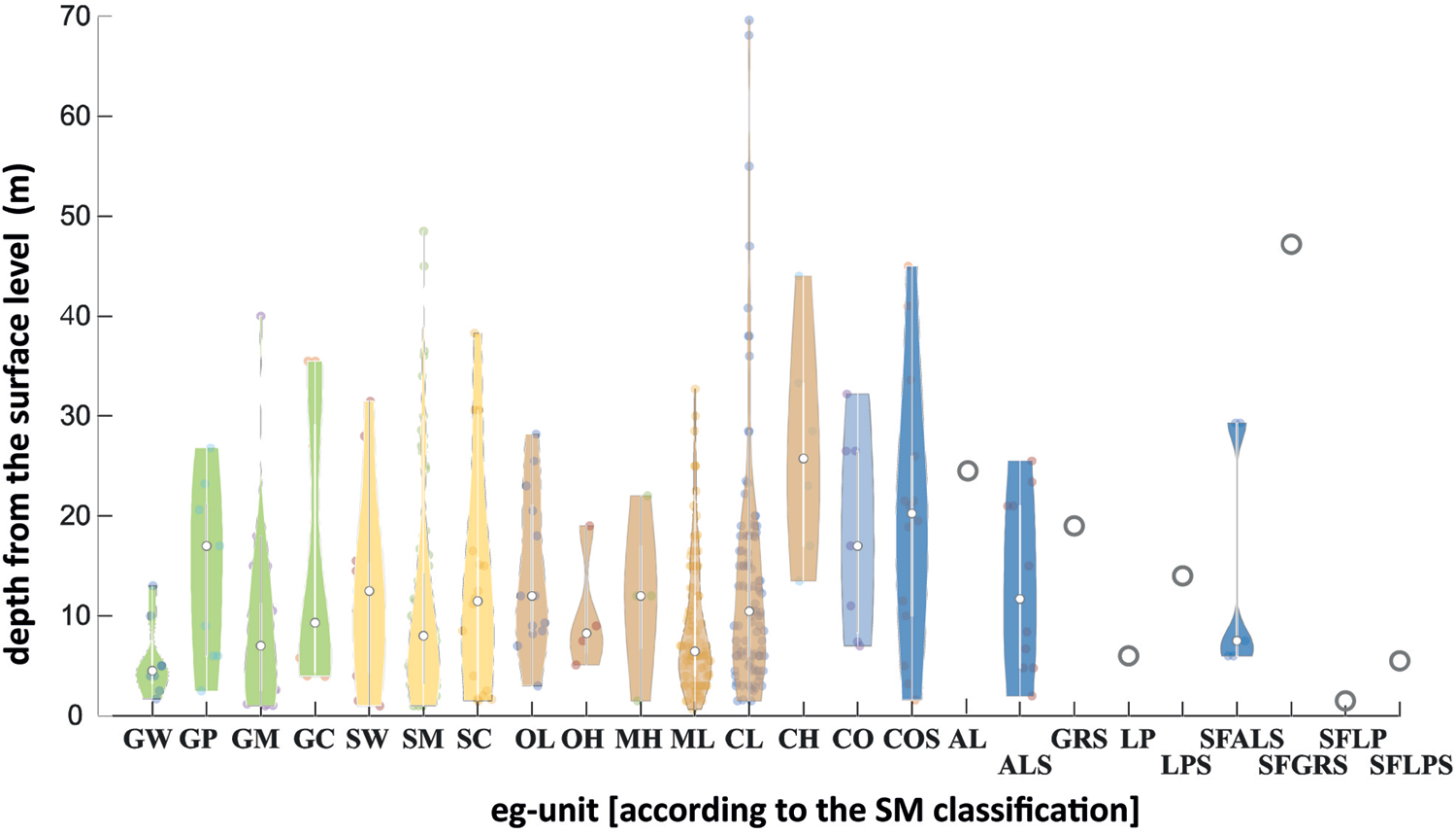
Violin plots for the depth of sampling for each eg-unit^53,54^.

Samples were taken mostly at depths ranging from 1 m to 70 m below ground level (Fig. 3).

**Fig. 4:**
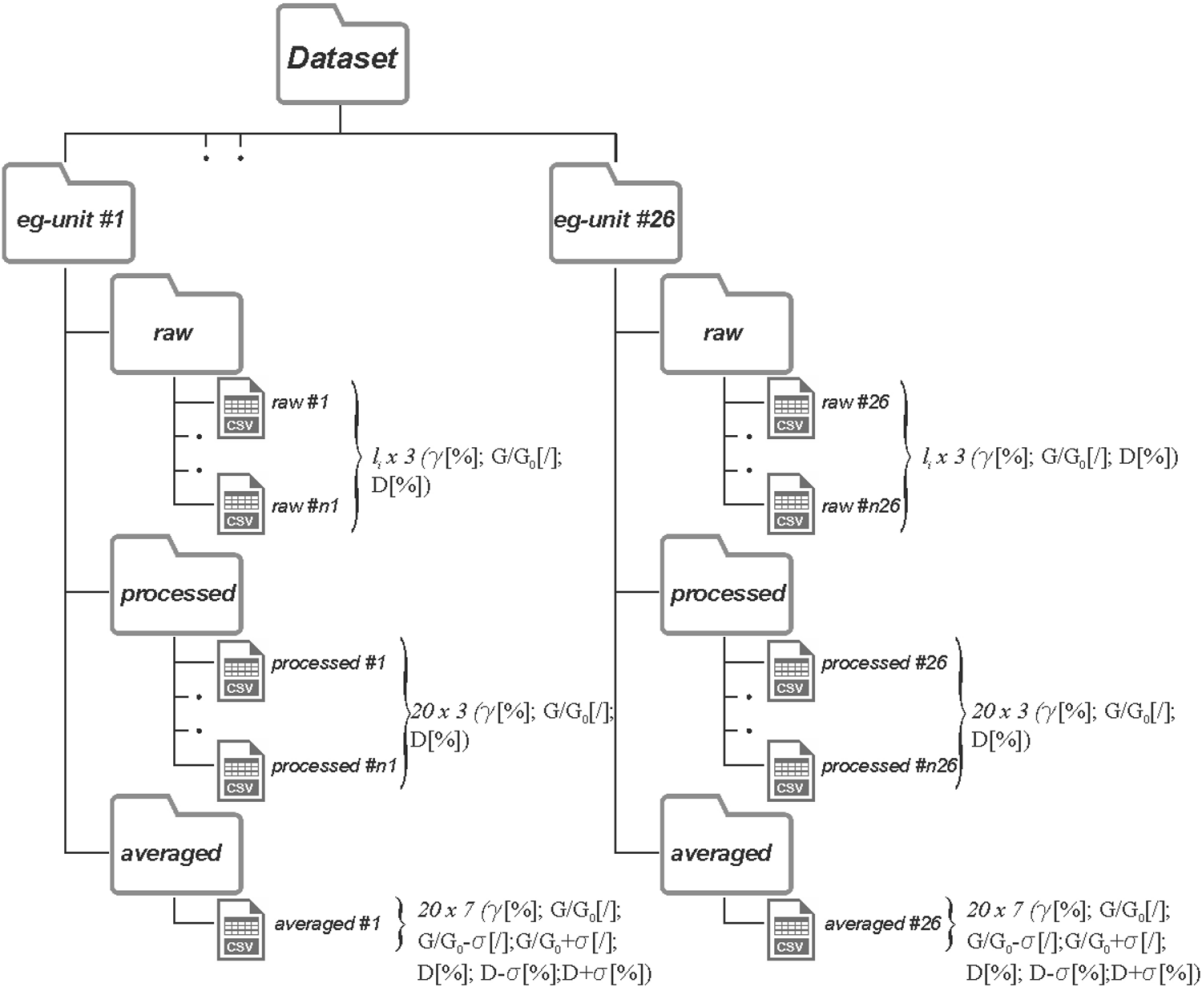
Depiction of the dataset structure (modified from Gaudiosi *et al*.^55^).

The initial (small strain) values of the damping ratio, D_0_, range between 0.2% and 63%. The smallest and the largest shear strain values obtained from laboratory tests are 1.0 × 10^−5^ % and 5.2 × 10^−1^ %, respectively. The dataset also includes some samples of a few organic clays with very high water content and low unit weight. The structure of the data array is depicted in Fig. 4. Moreover, each case history includes identifying information (e.g. ID, geographic coordinates) in a metadata file: the compiled post-processed data is presented in a single file suitably archived.

## Technical Validation

**Fig. 5:**
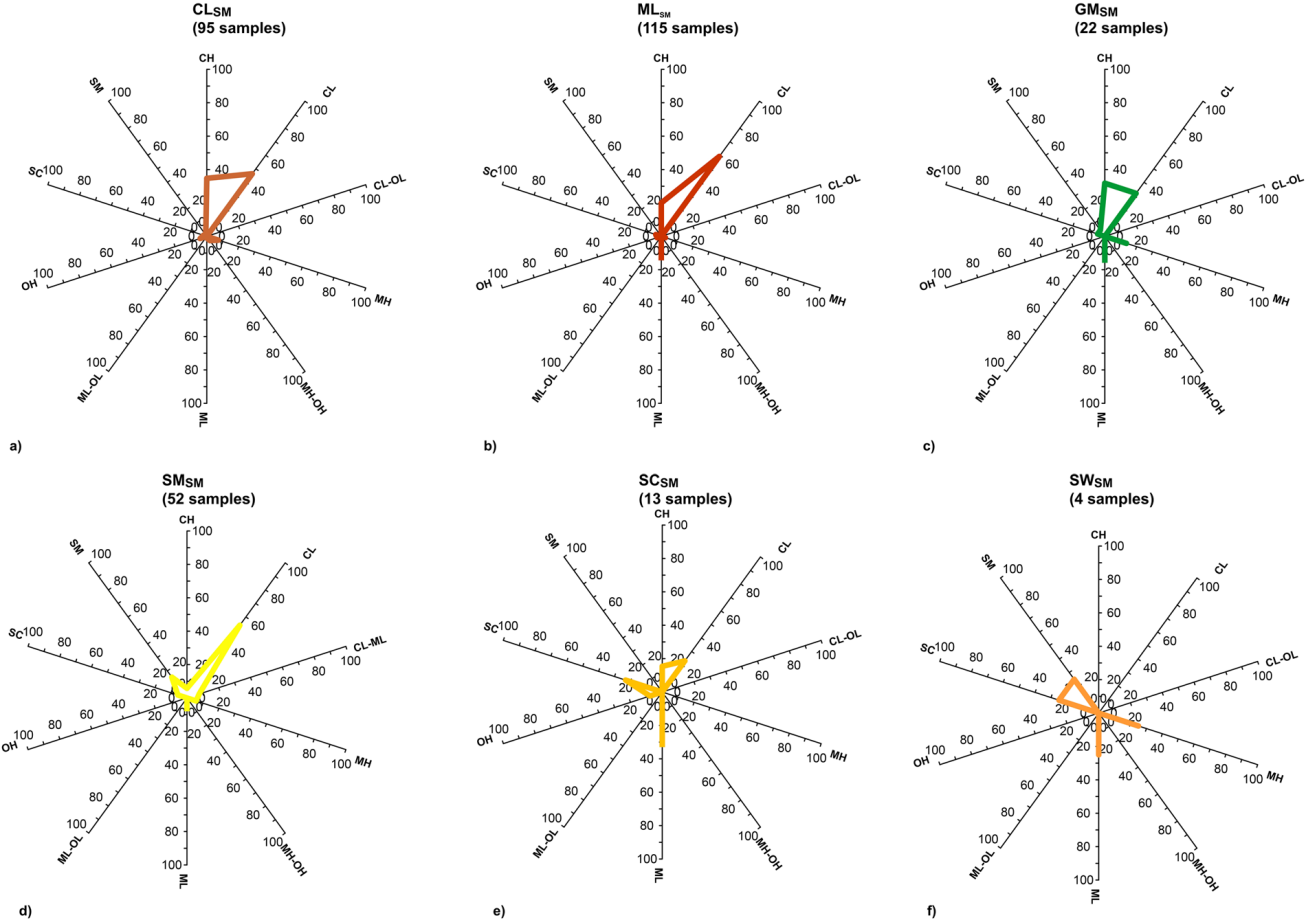
Vector distribution among USCS classes for the eg-units CL_*SM*_ (**a**), ML_*SM*_ (**b**), GM_*SM*_ (**c**), SM_*SM*_ (**d**), SC_*SM*_ (**e**), and SW_*SM*_ (**f**). Units on the vector axes are synched and expressed in %.

All the data was associated with an engineering geological unit and, if possible, with the USCS classification. At this point, the discussion deserves a focus on the representativeness of the samples, since differences exist between the USCS units obtained from the laboratory certificate, and the eg-units in the dataset. This constitutes a crucial aspect intrinsic to the process of extending results that are available for a few centimetres (i.e. the dimensions of the samples) to meters (i.e. the layer thickness) and from a few verticals (i.e. boreholes) to larger areas (i.e. cross-sections and seismically homogeneous microzones, SHM^8^). Statistical analysis was performed to investigate the correspondence between the USCS and eg-unit in all those cases where the two classifications are available for at least 7 samples. Figure 5 shows the distribution of the USCS codes among each seismic microzonation code as vectors going from the origin of the plot to the percentage of data availability (each angle direction is represented by a different USCS code).

**Fig. 6:**
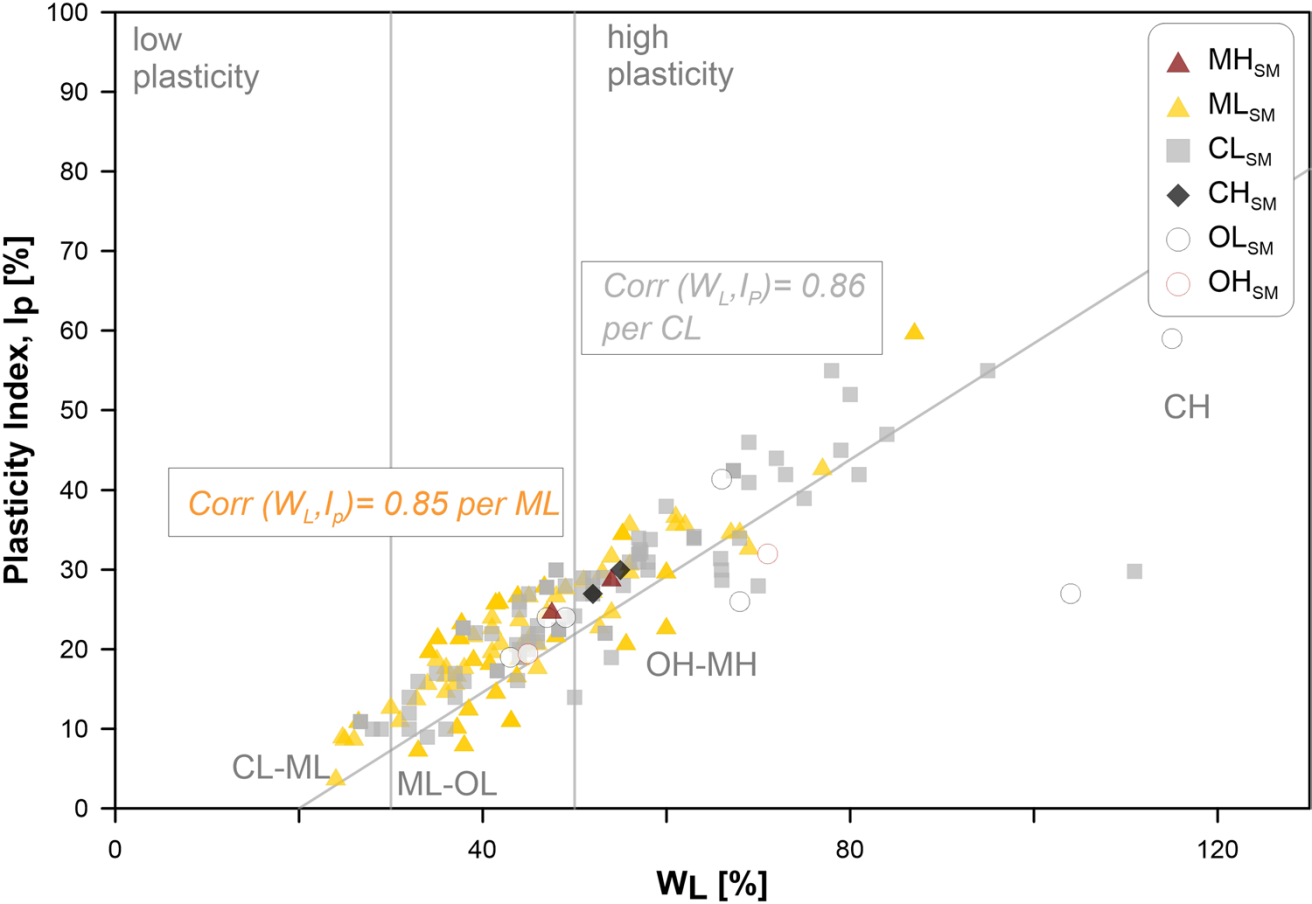
Scatter plot of the classification of the finer soils according to the Casagrande chart. SM subscript in legend stands for “seismic microzonation perspective”.

No code attributed in the seismic microzonation coincides with the USCS code for more than 50% of the population. From Fig. 5, it can be seen that for CL_*SM*_ only 46% of the samples (44 of a population of 95 samples) are described by the same acronym. Meanwhile, for ML_*SM*_ only 12% of the samples (14 out of 115 samples) are described by the same acronym, while for SM_*SM*_ only 15.4% of the samples (8 of 52 samples). The heterogeneity and anisotropy in the materials and geological formations seem more marked in the cases of SC_*SM*_ and GM_*SM*_ than in the cases of CL_*SM*_, ML_*SM*_ and SM_*SM*_. This behaviour may be due to the nature of the materials that constitute the specimens and to difficulties in the sampling operations: in all these cases, the specimens contain some finer levels of the main coarse deposits. In general, three concomitant aspects should be considered as sources of bias: 1) heterogeneity and anisotropy in the materials and geological formations; 2) unavailability of samples according to regular meshes of investigation, due to the cost of a theoretical massive-invasive exploitation; 3) subjectivity in the visual inspections of the sample. This latter may induce different attributions of the code attributed in SM. In the case of the finer materials, the differences between the two classifications were extrapolated also on a Casagrande chart for the most populated eg-unit classes (Fig. 6). Contextually, the correlation coefficients for CL_*SM*_ and ML_*SM*_ were also computed. The values (equal to 0.86 and 0.85, respectively) denote a higher variability in the case of ML_*SM*_ than CL_*SM*_ for the two variables: water content, WL and Plasticity Index, IP.

**Fig. 7:**
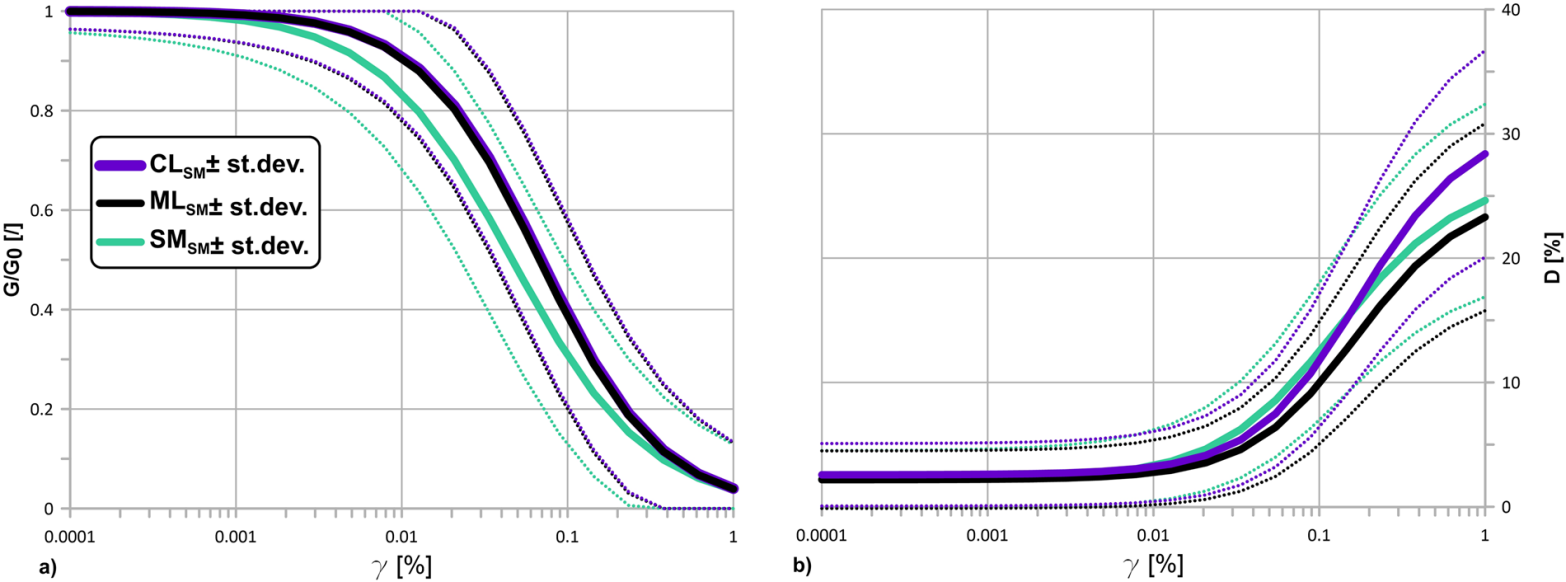
G\*G*_0_ (*γ*) curves (**a**) and D(*γ*) curves (**b**), adapted from Yokota *et al*.^26^, for *α*_*CLSM*_, *α*_*MLSM*_ and *α*_*SMSM*_ eg-units. Each unit is represented by the mean and by the Darendeli confidence levels (±95%)^37^.

As a further step, a representation of these in terms of median formulations of the G\*G*_0_(*γ*) and D(*γ*) curves were obtained (Fig. 7). The laws of variation of G\*G*_0_(*γ*) and D(*γ*) curves for each eg-unit were determined through the formulation of Darendeli^37^, which describes the standard deviation for the normalized modulus reduction and the damping curves in the form of equations based on statistically retrieved parameters. Mean and mean ± standard deviation curves for each unit were also made available in the archive in the folder “average”. The standard deviations have the form indicated in Eqs. 3, 4, respectively, for G\*G*_0_(*γ*) and D(*γ*):

3 $${\sigma }_{\frac{G}{{G}_{0}}}={e}_{13}^{\phi }+\sqrt{\frac{0.25}{{e}_{14}^{\phi }}-\frac{{\left(\frac{G}{{G}_{0}}-0.5\right)}^{2}}{{e}_{14}^{\phi }}}$$

4 $${\sigma }_{D}={e}_{15}^{\phi }\cdot {e}_{16}^{\phi }\cdot \sqrt{D}$$

where:

$${e}_{13}^{\phi }=-4.23$$

$${e}_{14}^{\phi }=3.62$$

$${e}_{15}^{\phi }=-5$$

$${e}_{16}^{\phi }=-0.25$$

In Table 2 the maximum and minimum values of the *σ*_*G\G*0_ and *σ*_*D*_ with the strain are reported for each eg-group. The values quantify the apparent aleatory randomness of the G\*G*_0_ and D values with a confidence level of 95%.

**Table 2:** Minimum and maximum *σ* values for each eg-group.

eg-unit	*min*_*σG*\*G*0_	*min*_*σD*_	*max*_*σG*\*G*0_	*max*_*σD*_
**AL**	0.03	1.76	0.10	3.05
**ALS**	0.02	1.46	0.10	3.72
**CH**	0.02	1.31	0.10	4.02
**CL**	0.02	1.26	0.10	4.16
**CO**	0.02	1.56	0.10	4.20
**COS**	0.02	1.23	0.10	4.56
**GC**	0.02	1.22	0.10	3.98
**GM**	0.02	1.34	0.10	3.55
**GP**	0.02	1.37	0.10	3.66
**GRS**	0.02	0.99	0.10	4.28
**GW**	0.02	1.38	0.10	4.05
**LPS**	0.02	1.77	0.10	3.25
**LP**	0.02	1.96	0.10	3.15
**MH**	0.02	1.13	0.10	3.92
**ML**	0.02	1.16	0.10	3.77
**OH**	0.02	1.11	0.10	3.41
**OL**	0.02	1.16	0.10	3.43
**SC**	0.02	1.21	0.10	3.32
**SFALS**	0.02	1.52	0.10	3.94
**SFLP**	0.02	1.18	0.10	3.32
**SFLPS**	0.02	1.22	0.10	3.94
**SM**	0.02	1.16	0.10	3.87
**SW**	0.02	1.20	0.10	3.21

Comparing the curves in terms of mean values, only significant differences for the SM_*SM*_ curves may be distinguished with respect to the ML_*SM*_ and CL_*SM*_ curves at low *γ* values (lower than 0.03%): the CL_*SM*_ and ML_*SM*_ eg-units follow approximately the same behaviour both for G\*G*_0_ and for D.

We tested the null hypothesis of the pairwise difference between data vectors of the *α* and *β* parameters used to smooth the curves according to Yokota *et al*.^26^. The results are synthetized in Table 3. At the 5% significance level, the returned value of h = 1 for *α*_*CLSM*_ vs *α*_*SM SM*_ indicates that the t-Student test rejects the null hypothesis, and thus suggests the presence of significant differences between the two populations. Symbol p in Table 2 is the probability of observing a test statistic to be as extreme as, or more extreme than, the observed value under the null hypothesis. For *α*_*MLSM*_ and *α*_*SM SM*_ the returned value of p is equal to 0.9; otherwise, for *α*_*CLSM*_ and *α*_*MLSM*_, p = 0.13. No significant differences are identified among the three populations of *β* used for D regularization.

**Table 3:** t-Student test.

	*α*_*CLSM*_ vs *α*_*MLSM*_	*α*_*CLSM*_ vs *α*_*SMSM*_	*α*_*MLSM*_ vs *α*_*SMSM*_
**p**	0.13	0.04	0.90
	*β*_*CLSM*_ vs *β*_*MLSM*_	*β*_*CLSM*_ vs *β*_*SMSM*_	*β*_*MLSM*_ vs *beta*_*SMSM*_
**p**	0.11	0.22	0.76

**Fig. 8:**
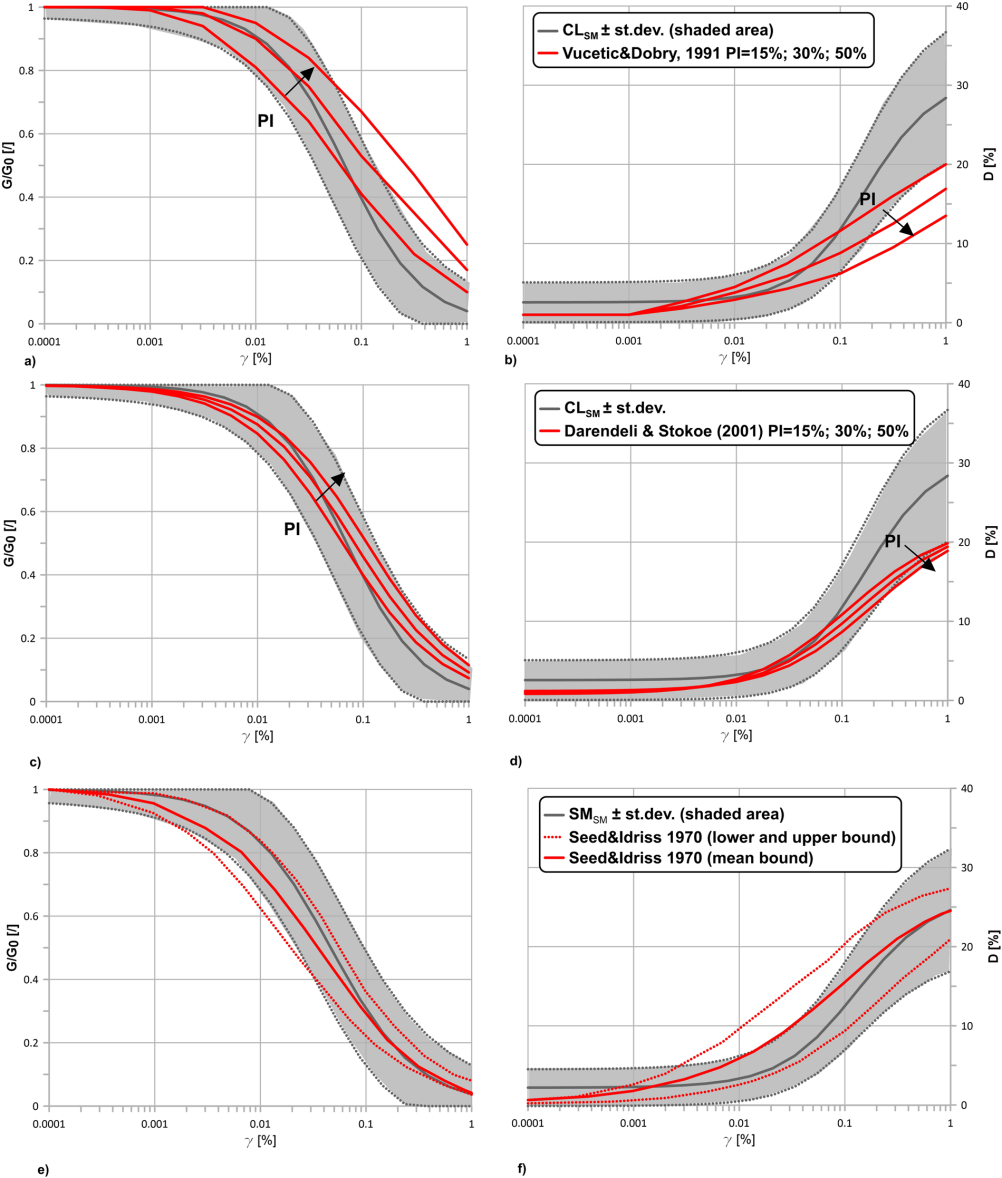
Comparisons with existing literature curves. G\*G*_0_(*γ*) and D(*γ*) curves for CL_*SM*_ eg-unit (**a** and **b,** **c** and **d**) ± Darendeli conference levels (adapted from Yokota *et al*.^26^), for a mean confining effective pressure *σ*’ of about 200 kPa, compared respectively with Vucetic and Dobry^39^ and Darendeli and Stokoe^40^, confining effective pressure *σ*’ = 200 kPa; G\*G*_0_(*γ*) and D(*γ*) curves for SM_*SM*_ eg-unit (**e** and **f**) ± Darendeli conference levels (adapted from Yokota *et al*.^26^), for a mean confining effective pressure *σ*’ of 180 kPa, compared with Seed and Idriss^56^ curves – mean, upper and lower bound.

As stated in Wasserstein *et al*.^38^, conclusions should not be based solely on whether an association was found to be statistically significant. According to this consideration, the most commonly used in numerical modelling curves were investigated. The variation with the Vucetic and Dobry^39^ and Darendeli and Stokoe^40^ models were simulated for a Plasticity Index ranging from 15 to 50% (Fig. 8). The highest differences of the means curves for CL_*SM*_ from those in the literature may be recorded at very high strain levels (0.3 and 0.8–0.9% respectively for G\*G*_0_ and D). Generally, the behaviour of the seismic microzonation curves ± the standard deviations is able to include the predicted variability based on the Plasticity Index recorded in the dataset. Despite this evidence, the SM_*SM*_ curves show the highest standard deviations compared to the literature data.

As a remark, the rationale of this study is that by extending seismic microzonation data, it is possible to account for uncertainty in a coherent framework, where subsurface geometries and buried morphologies also have a similar amount of uncertainty. As a result, the predictions made by this study are larger than those in the literature are.

## Usage Notes

**Fig. 9:**
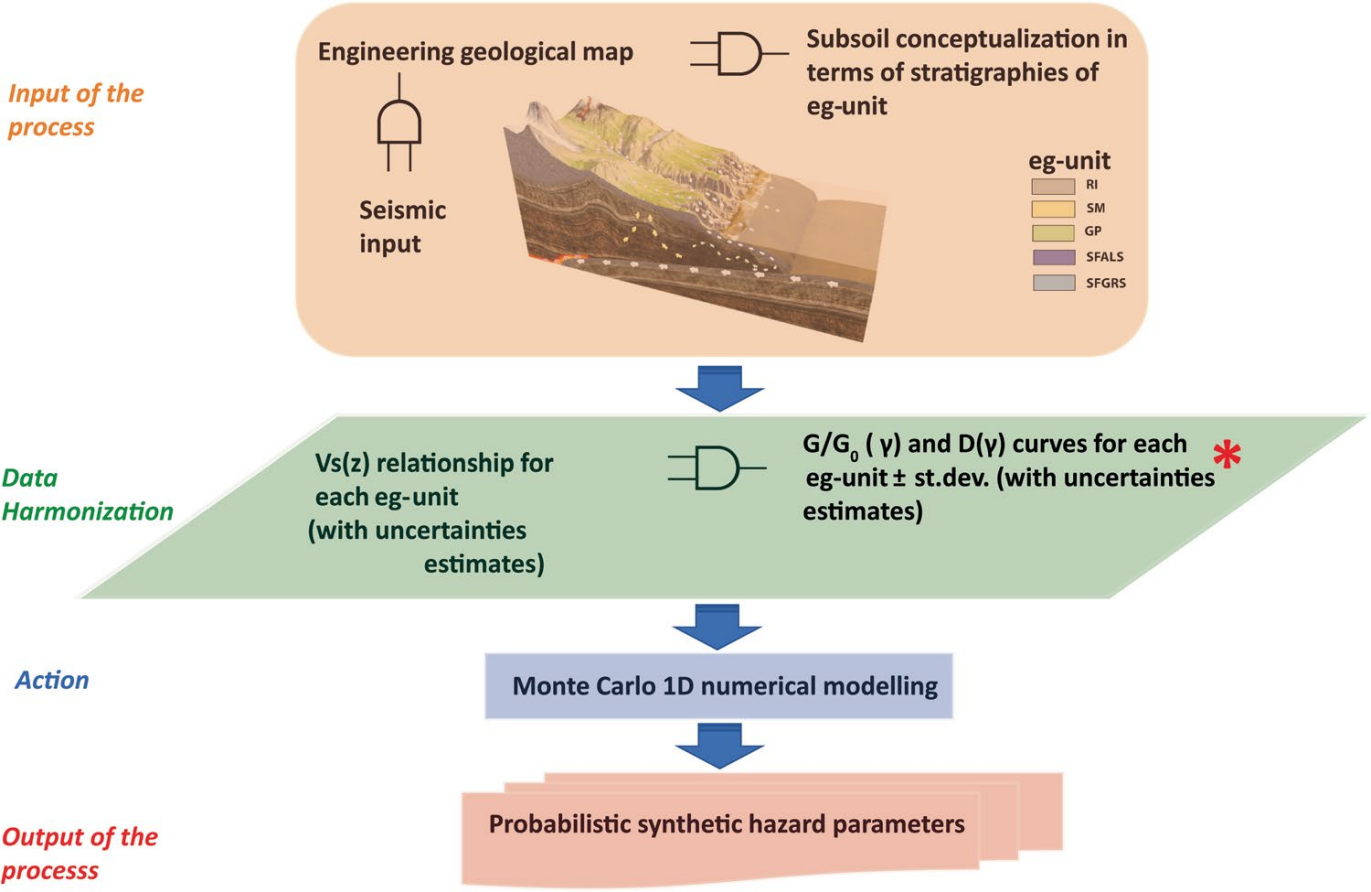
Flowchart of the overall process devoted to the probabilistic hazard assessment. Red asterisk indicates the present research positioning.

The curves shown in this work and identified using the laboratory data of the seismic microzonation studies can be adopted as input in 1D calculation codes to carry out local seismic response studies, as shown in Fig. 9.

The results shown before suggest that a further merge of the eg-units is possible. This was previously confirmed also in terms of the S-waves velocity, Vs by Romagnoli *et al*.^19^. In practice, from the point of view of the non-linear behaviour of soils, a macro-group of eg-units may be constructed including all the eg-units relating to clays and inorganic silts (ML, CL, MH and CH) in one single macro-group, while two other macro-groups may be defined for: 2. OH + OL and 3. SM + SC + SP + SW. It is worth noticing that all the curves defined for each macro-group may be adopted only to reproduce the response of soils located whitin the first 15 m. The laws of variations of G\*G*_0_ and D have the forms indicated by Eqs. 5, 6, respectively, and the parameters of Table 4.

5 $$\frac{G}{{G}_{0}}\left(\gamma \right)={a}_{1}\cdot {e}^{{b}_{1}\cdot \gamma }+{c}_{1}\cdot {e}^{{d}_{1}\cdot \gamma }$$

6 $$D\left(\gamma \right)={a}_{2}\cdot {e}^{{b}_{2}\cdot \gamma }+{c}_{2}\cdot {e}^{{d}_{2}\cdot \gamma }$$

**Table 4:** Coefficients (with 95% confidence bounds) for the aggregated formulations for the macro-groups: 1. ML + CL + MH + CH (mean confining effective pressure *σ*’ = 250 kPa); 2.

G\*G*_0_ (*γ*)		
1. ML + CL + MH + CH	2. OH + OL	3. SM + SC + SP + SW
Coefficients (with 95% confidence bounds):	Coefficients (with 95% confidence bounds):	Coefficients (with 95% confidence bounds):
a1 = 0.061 (0.058, 0.063)	a1 = 0.078 (0.07303, 0.08331)	a1 = 0.0602 (0.058, 0.063)
b1 = −0.924 (−1.062, −0.785)	b1 = −0.247 (−0.377, −0.117)	b1 = −0.769 (−0.903, −0.635)
c1 = −0.053 (−0.056, −0.051)	c1 = −0.067 (−0.072, −0.061)	c1 = −0.051 (−0.054, −0.049)
d1 = −76.690 (−88.350, −65.020)	d1 = −30.990 (−37.310, −24.670)	d1 = −84.280 (−98.320, −70.230)
**D (*γ*)**		
**1. ML + CL + MH + CH**	**2. OH + OL**	**3. SM + SC + SP + SW**
Coefficients (with 95% confidence bounds):	Coefficients (with 95% confidence bounds):	Coefficients (with 95% confidence bounds):
a2 = 2.942 (2.891, 2.994)	a2 = 3.047 (3.022, 3.072)	a2 = 2.074 (2.030, 2.119)
b2 = 0.124 (0.102, 0.147)	b2 = 0.126 (0.118, 0.134)	b2 = 0.182 (0.155, 0.2094)
c2 = −2.213 (−2.264, −2.162)	c2 = −1.537 (−1.561, −1.512)	c2 = −1.795 (−1.839, −1.752)
d2 = −8.570 (−8.922, −8.218)	d2 = −3.774 (−3.843, −3.705)	d2 = −8.169 (−8.524, −7.813)

OH + OL (mean confining effective pressure *σ*’ = 150 kPa); 3.SM + SC + SP + SW (mean confining effective pressure *σ*’ = 170 kPa).

The G\*G*_0_ and D curves may be described using the aggregated variation laws defined ad hoc for seismic microzonation through the parameters reported in Table 4. For completeness, Table 5 reports also the maximum and minimum values of the *σ* for G\*G*_0_ and D with the strain for the three previously introduced macro-groups.

**Table 5:** Minimum and maximum *σ* values for G\*G*_0_ and D for the macro-groups: 1.

*min*_*σG*\*G*0_		
1. ML + CL + MH + CH	2. OH + OL	3. SM + SC + SP + SW
0.0055	0.0077	0.0088
***max***_**σG\G0**_		
**1. ML + CL + MH + CH**	**2. OH + OL**	**3. SM + SC + SP + SW**
0.058	0.077	0.061
***min***_***σD***_		
**1. ML + CL + MH + CH**	**2. OH + OL**	**3. SM + SC + SP + SW**
0.7402	1.5124	0.2803
***max***_***σD***_		
**1. ML + CL + MH + CH**	**2. OH + OL**	**3. SM + SC + SP + SW**
3.322	3.421	2.478

ML + CL + MH + CH; 2. OH + OL; 3.SM + SC + SP + SW.

Thus, the parameters of the hyperbolic model for eg-unit groups and macro groups, respectively, are shown in Tables 2, 5. Neverthless, the outcomes of this study can be used in any code that simulates 1D propagating waves by using the parameters provided in Table 4 and the formulation in Eqs. (5, 6), when Darendeli’s model is not implemented.

The present work fits in the field of fully probabilistic seismic hazard assessment. The level at which these results feature in the entire process is indicated in Fig. 9 with a red asterisk.

It is outside the scope of the work to suggest a correlation model that examines the connection between the variation in G\*G*_0_ reduction and the variation in D increase^41,42^, but it may be a topic for future research.

The dataset may be used to adapt models from the laboratory to the regional/local scale, similarly to what happens for analogous models in the laboratory to real-scale models^43^. In other words, the eg-unit definition allows the modelling of the dynamical properties of a geological body when changes of scale are applied. This scaling operation is then even more important considering that at least four sources of uncertainties may affect the numerical modelling results when using laboratory tests data as inputs: 1) loading directionality; 2) simplified schemes of application of the cyclic loading; 3) drainage conditions and 4) representativeness of samples. The present lack of knowledge of engineering geology at a regional scale has until now limited the interpretation of the available data. Thus, these results aim to provide new insights about this topic, consequentially looking at seismic prevention at a regional scale, rather than at single municipality scale. This scale is even more important since agglomerates of adjacent hamlets strictly interact with each other. This approach is one of the principles at the base of the new Italian code of Civil Protection^44^.

Moreover, this study illustrates relevant information in the perspective of performing 3D numerical modelling at a local/regional scale, which was described as one of the grand challenges by Forsyth *et al*.^45^. 3D numerical modelling is recently being even more widely diffused and adopted because of its ability to explain the complex pattern of strong ground motions after or before an earthquake event, but nowadays only linear simulations are performed due to the computational cost and lack of data. Therefore, starting from the average values of the defined curves of this study, future simulations could be run where eg-unit models are available^46,47^.

The cascade effect resulting from this analysis can also provide new data suitable for achieving a detailed physical understanding of the nonlinear processes of waves propagation after events causing damage. It is generally assumed as a rule of thumb that the damping ratio D may be related to the a-dimensional Q factor using the expression: D = 1\ (2Q)^48^. On this subject, Dimitriu *et al*.^49^ and Lacave-Lachet *et al*.^50^ also showed that an important contribution to *κ* (the seismological measure of wave attenuation) is the inelastic attenuation (D) in the site’s subsurface geology. The data described in this study can provide further information suitable for comparisons with seismological data^51^ and it consequentially has the potential to be used for several purposes (i.e., stochastic ground-motion prediction calculations; nonlinearity and attenuation of seismic waves relationships).

Moreover, the present dataset may allow the relaxing of the ergodicity hypothesis on the nonlinearity among all the parameters, which regulates the seismic response which also includes stratigraphy, shear wave velocities and Vs.

## Data Availability

Matlab code was used to generate regularization and statistics. RawGraphs was used to realize Fig. 2 (see Mauri *et al*.^52^).
